# Differential white blood cell counts in rabbits: a comparison of the Advia 2120 and a manual method

**DOI:** 10.1177/10406387211007877

**Published:** 2021-04-09

**Authors:** Ioannis L. Oikonomidis, Elspeth Milne, Chiara Piccinelli

**Affiliations:** Easter Bush Pathology, Royal (Dick) School of Veterinary Studies and The Roslin Institute, University of Edinburgh, Roslin, UK

**Keywords:** blood smear, complete blood count, hematology, leporine, leukocyte

## Abstract

We evaluated the performance of the Advia 2120 (Siemens) differential leukocyte count (A-Diff) compared to the manual method (M-Diff) in rabbits. EDTA-anticoagulated blood samples collected for diagnostic purposes were analyzed within 6 h of collection. The M-Diff was performed blindly by 2 observers on blood smears by counting 200 cells. We initially included 117 samples; 25 samples were excluded because of suboptimal gating of leukocytes in the Advia peroxidase cytogram or poor blood smear quality. The correlation between the A-Diff and M-Diff was very high for heterophils (r = 0.924, *p* < 0.001) and lymphocytes (r = 0.903, *p* < 0.001), high for basophils (r = 0.823, *p* < 0.001), moderate for monocytes (r = 0.645, *p* < 0.001), and low for eosinophils (r = 0.336, *p* = 0.001). The Passing–Bablok regression analyses revealed a small-to-moderate constant error for lymphocytes and a slight constant error for basophils. Small proportional errors were detected for heterophils, lymphocytes, and eosinophils. The Bland–Altman analyses revealed that the Advia significantly underestimates heterophils and overestimates lymphocytes compared to M-Diff. The biases for the other leukocytes were minimal and likely clinical insignificant; however, our results, particularly for eosinophils, should be interpreted cautiously given the observed low percentages in our samples. Given the observed biases in heterophil and lymphocyte percentages in the Advia 2120 CBC results in rabbits, method-specific reference intervals should be used. The Advia can recognize leporine basophils. Evaluation of blood smears is still recommended to investigate abnormal results and erroneous cytograms reported by the Advia.

The complete blood count (CBC) is an essential part of the diagnostic investigation of domestic animals. It is typically performed with the use of automated hematology analyzers equipped with multispecies software. Nevertheless, a manual differential leukocyte count (M-Diff) still commonly complements the CBC in veterinary medicine. On the contrary, in human medicine, the M-Diff has been largely replaced by the automated differential leukocyte count (A-Diff) for routine hematologic investigation, given that the new automated hematology analyzers are generally considered highly accurate, whereas the M-diff is laborious and inherently imprecise.^7,12^

The Advia 2120 hematology system (Siemens) is a laser-based hematology analyzer that is equipped with multispecies software. The Advia 2120 and its precursor, the Advia 120, are commonly used in veterinary laboratories, veterinary hospitals, and pharmaceutical companies. The Advia has been validated previously for the measurement of reticulocytes in rabbits^8^; its ability to recognize leporine basophils has also been studied using a limited number of blood samples with basophilia.^10^ However, to our knowledge, neither the Advia 2120 nor the Advia 120 has been validated previously for determining the 5-part differential leukocyte count in rabbits. Rabbits are becoming increasingly popular as pets,^14^ and are also commonly used as an animal model for various human diseases^3^; therefore, a study of the performance of the Advia in determining the differential leukocyte count in rabbits is needed.

We compared the A-Diff provided by the Advia 2120 to the M-Diff in rabbits. We hypothesized that the Advia 2120 A-Diff would be suitable for use in rabbits.

## Materials and methods

We used blood samples from rabbits collected into 0.5-mL tubes containing K_3_EDTA as anticoagulant (Teklab) for diagnostic purposes in a veterinary teaching hospital between March 2018 and May 2019. The samples reflected the general variability of patients admitted to a first opinion and referral center, ranging from health checks to hospitalized patients. CBCs were performed on the Advia 2120 with species-specific software within 6 h of blood collection, and blood smears were made in the same timeframe. The M-Diffs were performed by 2 independent observers [a 3rd-year resident in clinical pathology (I. Oikonomidis) and a board-certified clinical pathologist (C. Piccinelli)] on modified Wright-stained blood smears by counting 200 cells. The observers were blinded to the Advia 2120 results. The M-Diffs were done within the monolayer of the blood smear by moving in a zig-zag pattern to avoid covering the same area of the slide twice.^5^ A modification of a previously described semi-quantitative scoring system^5^ was used to report the presence and severity of toxic changes in heterophils (Table 1). The presence, number, and size of platelet clumps were assessed using a semi-quantitative scoring system described previously,^17^ with slight simplification (Table 1). The samples were excluded from the study when one of the following criteria was met: 1) under- or over-filled EDTA tubes; 2) samples with visible clots; 3) samples with poor differentiation of leukocyte clusters on Advia peroxidase (PEROX) cytograms (visual inspection was performed by the same 2 observers); and 4) samples with blood smears of poor quality (i.e., presence of many lysed WBCs, suboptimal distribution of the WBCs throughout the smear, or frequent trapping of leukocytes in platelet aggregates).

**Table 1.** Scoring system for the presence and severity of toxic changes in heterophils and platelet clumping in blood smears of rabbits. A modification of a previously described semi-quantitative scoring system^5^ was used to report the presence and severity of toxic changes in heterophils. The presence, number, and size of platelet clumps were assessed using a previously described semi-quantitative scoring system^17^ with slight simplification.

Grade	Proportion of heterophils with toxic changes (%)	Severity of cytoplasmic toxic changes	No. and size of platelet aggregates in the blood smear
0	< 5	Absence of toxic changes	Absence of platelet aggregates
1	5–10	A few dark-purple cytoplasmic granules	< 5 small aggregates
2	11–30	Mildly decreased numbers of normal brick-red staining cytoplasmic granules; low-to-moderate numbers of dark-purple cytoplasmic granules; mild cytoplasmic basophilia	> 5 small aggregates or 1–2 large aggregates
3	> 30	Moderately to markedly decreased numbers of normal brick-red staining cytoplasmic granules; frequent dark-purple cytoplasmic granules; moderate-to-marked cytoplasmic basophilia	≥ 3 large aggregates

Small aggregates = 5–20 platelets; large aggregates = > 50 platelets.

The data distribution was assessed using the Shapiro–Wilk test. Depending on the data distribution, Pearson or Spearman correlation coefficients were used to correlate the results of the M-Diffs between the 2 observers, as well as the results of the A-Diff with those of the M-Diff (the mean of the 2 observers’ values were utilized for the latter). Passing–Bablok regression analysis and Bland–Altman analysis were employed to evaluate the performance of the A-Diff compared to the M-Diff according to the most recent American Society for Veterinary Clinical Pathology guidelines for method comparison.^1^ Statistical analyses were performed using the statistical language R (https://www.r-project.org/).

## Results

We initially included 117 leporine samples in our study; 13 samples were excluded because the stained blood smears were considered of poor quality. An initial statistical analysis was performed using the data from the remaining 104 samples (Table 2, Suppl. Table 1). After evaluating the Advia 2120 results, 12 samples were excluded because of suboptimal gating in the PEROX cytogram that resulted in indistinct differentiation of heterophils, eosinophils, lymphocytes, and monocytes. No overt morphologic abnormalities were detected in 11 of 12 blood smears of the samples that were excluded; in one sample, some karyorrhectic or pyknotic cells were noted. We eventually included 92 samples for analysis.

**Table 2.** Results of the Passing–Bablok and Bland–Altman analyses comparing the differential leukocyte counts obtained by the Advia 2120 and the manual method in 92 leporine blood samples. Thirteen samples were excluded previously from analysis because of poor blood smear quality and another 12 samples were excluded because of suboptimal gating of leukocytes in the Advia peroxidase cytogram. The manual differential leukocyte counts were performed by 2 blinded, independent observers by counting 200 cells in modified Wright-stained blood smears. The mean values obtained from the 2 observers were utilized for the statistical analysis.

Leukocyte	Bias	Lower limit of bias	Upper limit of bias	Estimated intercept	Estimated slope
Heterophils	−9.3 (−10.6, −8.1)	−21.5 (−23.7, −19.3)	2.8 (0.5, 5.0)	−3.62 (−7.24, 0.65)	0.91 (0.82, 0.98)
Lymphocytes	5.5 (4.0, 6.9)	−8.4 (−10.9, −5.9)	19.3 (16.8, 21.8)	7.17 (3.80, 11.09)	0.93 (0.85, 0.99)
Monocytes	1.5 (1.0, 2.0)	−3.4 (−4.2, −2.5)	6.3 (5.4, 7.2)	0.77 (−0.23, 1.85)	1.08 (0.92, 1.32)
Eosinophils	1.3 (1.1, 1.6)	−1.2 (−1.7, −0.7)	3.8 (3.4, 4.3)	0.53 (−0.35, 0.80)	2.20 (1.40, 4.41)
Basophils	0.7 (0.4, 1.0)	−2.3 (−2.8, −1.7)	3.6 (1.0, 3.1)	0.75 (0.45, 1.10)	0.93 (0.80, 1.06)

Numbers in parentheses are 95% confidence intervals.

The data distribution was Gaussian for heterophils and lymphocytes, and non-Gaussian for monocytes, eosinophils, and basophils. The mean (± SD) heterophil and lymphocyte percentages obtained by the Advia 2120 were 48.1 ± 14.9% and 39.1 ± 15.3%, respectively; the median (range) of monocyte, eosinophil, and basophil percentages were 5.5% (0.9–15.1%), 1.6% (0.3–10.5%), and 4.0% (0.5–10.0%), respectively (Suppl. Table 2). The median (range) of the large unstained cell (LUC) percentage was 0.4% (0–2.0%). The mean (± SD) heterophil and lymphocyte percentages obtained by the manual method were 57.5 ± 16.2% and 33.7 ± 16.5%, respectively; the median (range) of monocyte, eosinophil, and basophil percentages were 4.0% (0–17.0%), 0.5% (0–9.0%), and 3.3% (0–11.0%), respectively. The correlation between the manual counts performed by the 2 independent observers was very high for heterophils (r = 0.962, *p* < 0.001) and lymphocytes (r = 0.963, *p* < 0.001), high for monocytes (r = 0.800, *p* < 0.001) and basophils (r = 0.799, *p* < 0.001), and moderate for eosinophils (r = 0.584, *p* < 0.001). The correlation between A-Diff and M-Diff was very high for heterophils (r = 0.924, *p* < 0.001) and lymphocytes (r = 0.903, *p* < 0.001), high for basophils (r = 0.823, *p* < 0.001), moderate for monocytes (r = 0.645, *p* < 0.001), and low for eosinophils (r = 0.336, *p* = 0.001).

**Figure 1.**
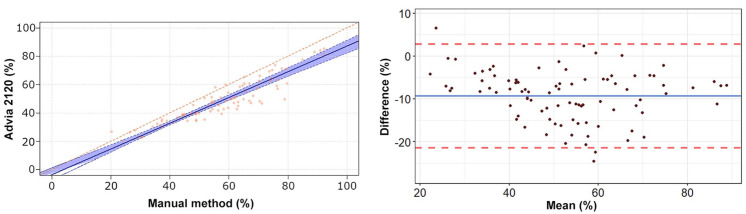
Passing–Bablok regression analysis and Bland–Altman plots of heterophil percentage obtained by the Advia 2120 compared to the manual method in 92 blood samples from rabbits. **Left.** The red diagonal line in the Passing–Bablok regression analysis plot is the line of identity, and the blue line is the calculated line of regression. The light blue area represents the 95% confidence intervals (CIs). **Right.** In the Bland–Altman plot, the difference between the 0 line and the blue line indicates the bias of the Advia 2120 minus the manual differential counts. The 95% CIs of the calculated bias are represented by the 2 red dashed lines. The manual differential leukocyte counts were performed by 2 blinded, independent observers by counting 200 cells in modified Wright-stained blood smears. The mean values obtained from the 2 observers were utilized for the statistical analysis.

**Figure 2.**
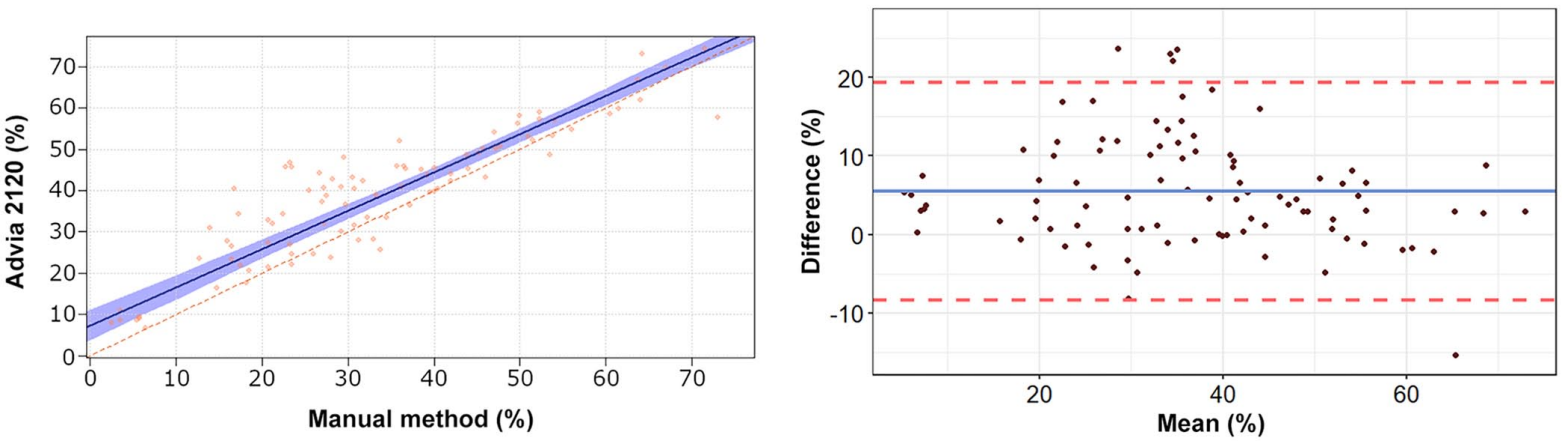
Passing–Bablok regression analysis and Bland–Altman plots of lymphocyte percentage obtained by the Advia 2120 compared to the manual method in 92 blood samples from rabbits. For detailed explanation of the plots, see the legend of Figure 1.

**Figure 3.**
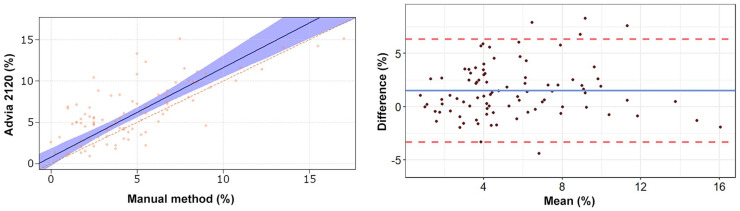
Passing–Bablok regression analysis and Bland–Altman plots of monocyte percentage obtained by the Advia 2120 compared to the manual method in 92 blood samples from rabbits. For detailed explanation of the plots, see the legend of Figure 1.

**Figure 4.**
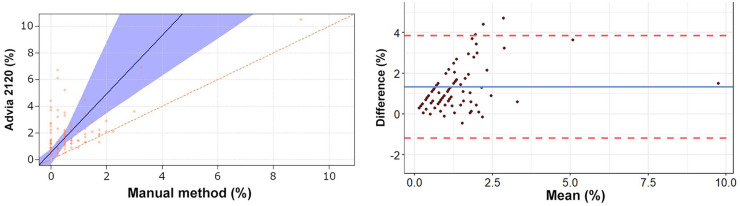
Passing–Bablok regression analysis and Bland–Altman plots of eosinophil percentage obtained by the Advia 2120 compared to the manual method in 92 blood samples from rabbits. For detailed explanation of the plots, see the legend of Figure 1.

**Figure 5.**
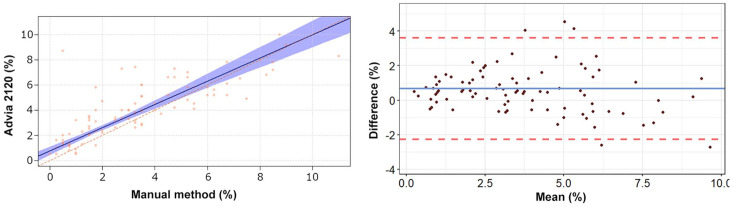
Passing–Bablok regression analysis and Bland–Altman plots of basophil percentage obtained by the Advia 2120 compared to the manual method in 92 blood samples from rabbits. For detailed explanation of the plots, see the legend of Figure 1.

The Passing–Bablok regression analyses revealed a statistically significant constant error for lymphocytes and basophils, and a statistically significant proportional error for heterophils, lymphocytes, and eosinophils (Table 2, Figs. 1–5). The Bland–Altman analyses revealed a statistically significant negative bias of −9.4% for heterophils and statistically significant positive biases of 5.5%, 1.5%, 1.3%, and 0.7% for lymphocytes, monocytes, eosinophils, and basophils, respectively, with wide 95% limits of agreement for heterophils and lymphocytes (Table 2, Figs. 1–5). On Bland–Altman plots, the difference between A-Diff and M-Diff was outside the calculated 95% confidence intervals in 3 of 92 cases for heterophils and eosinophils, and in 5 of 92 cases for lymphocytes, monocytes, and basophils.

Toxic changes in heterophils were observed in 36 of 92 (39%) samples. The proportion of heterophils with toxic changes was low (score 1) in 16 of 36 (44%) samples, moderate (score 2) in 13 of 36 (36%) samples, and high (score 3) in 7 of 36 (19%) samples. The severity of toxic changes was considered mild (score 1) in 32 of 36 (89%) samples and moderate (score 2) in 4 of 36 (11%) samples. After excluding the samples with toxic changes in heterophils, the correlation between A-Diff and M-Diff was very high for heterophils (r = 0.913, *p* < 0.001), high to very high for lymphocytes (r = 0.886, *p* < 0.001), high for basophils (r = 0.722, *p* < 0.001), moderate for monocytes (r = 0.555, *p* < 0.001), and low for eosinophils (r = 0.259, *p* = 0.054). Repeating the Passing–Bablok and Bland–Altman analyses after exclusion of samples with toxic changes in heterophils yielded results similar to the initial analyses (Table 3). Low numbers of reactive lymphocytes were noted in 21 of 92 (23%) blood smears.

**Table 3.** Results of the Passing–Bablok and Bland–Altman analyses comparing the differential leukocyte counts obtained by the Advia 2120 and the manual method in 56 leporine blood samples in which no evidence for toxic changes in heterophils was observed. The manual differential leukocyte counts were performed by 2 blinded, independent observers by counting 200 cells in modified Wright-stained blood smears. The mean values obtained from the 2 observers were utilized for the statistical analysis.

Leukocyte	Bias	Lower limit of bias	Upper limit of bias	Estimated intercept	Estimated slope
Heterophils	−9.2 (−11.0, −7.5)	−21.8 (−24.8, −18.8)	3.3 (0.4, 6.3)	−1.35 (−6.61, 6.51)	0.85 (0.70, 0.98)
Lymphocytes	4.7 (2.8, 6.7)	−9.6 (−13.0, −6.2)	19.0 (15.6, 22.4)	8.27 (3.20, 14.80)	0.89 (0.75, 0.99)
Monocytes	1.7 (1.0, 2.4)	−3.4 (−4.7, −2.2)	6.9 (5.7, 8.1)	0.54 (−0.83, 2.63)	1.14 (0.87, 1.58)
Eosinophils	1.6 (1.3, 2.0)	−1.2 (−1.8, −0.5)	4.4 (3.8, 5.1)	0.50 (−1.60, 1.15)	2.60 (1.20, 9.81)
Basophils	0.7 (0.3, 1.2)	−2.7 (−3.5, −1.9)	4.2 (3.4, 5.0)	0.72 (0.21, 1.30)	0.93 (0.77, 1.16)

Numbers in parentheses are 95% confidence intervals.

Platelet clumping was noted in 65 of 92 samples; it was considered mild (score 1) in 22 of 65 (34%) samples, moderate (score 2) in 10 of 65 (15%) samples, and marked (score 3) in 33 of 65 (51%) samples. After excluding the samples with marked platelet clumping in the blood smear, the correlation between A-Diff and M-Diff was very high for heterophils (r = 0.929, *p* < 0.001), high to very high for lymphocytes (r = 0.895, *p* < 0.001), high for basophils (r = 0.821, *p* < 0.001), moderate for monocytes (r = 0.532, *p* < 0.001), and low for eosinophils (r = 0.489, *p* = 0.001). Repeating the Passing–Bablok and Bland–Altman analyses after exclusion of samples with marked platelet clumping yielded results similar to the initial analyses (Table 4).

**Table 4.** Results of the Passing–Bablok and Bland–Altman analyses comparing the differential leukocyte counts obtained by the Advia 2120 and the manual method in 59 leporine blood samples in which no evidence for marked platelet clumping was observed. The manual differential leukocyte counts were performed by 2 blinded, independent observers by counting 200 cells in modified Wright-stained blood smears. The mean values obtained from the 2 observers were utilized for the statistical analysis.

Leukocyte	Bias	Lower limit of bias	Upper limit of bias	Estimated intercept	Estimated slope
Heterophils	−9.5 (−11.0, −8.0)	−20.9 (−23.5, −18.3)	2.8 (−0.8, 4.5)	−3.96 (−10.19, 1.82)	0.91 (0.80, 1.03)
Lymphocytes	5.6 (3.7, 7.4)	−8.3 (−11.5, −5.1)	19.4 (16.2, 22.6)	7.27 (3.10, 13.42)	0.93 (0.79, 1.02)
Monocytes	1.8 (1.1, 2.5)	−3.4 (−4.6, −2.2)	7.1 (5.9, 8.3)	0.28 (−1.07, 2.47)	1.23 (0.89, 1.65)
Eosinophils	1.1 (0.8, 1.4)	−1.1 (−1.6, −0.6)	3.3 (2.8, 3.8)	0.50 (0.03, 0.90)	1.60 (1.07, 3.20)
Basophils	0.5 (0.1, 0.9)	−2.5 (−3.2, −1.8)	3.6 (2.9, 4.2)	0.66 (0.35, 1.00)	0.88 (0.78, 1.04)

Numbers in parentheses are 95% confidence intervals.

## Discussion

To our knowledge, the performance of the Advia 2120 5-part differential leukocyte count in rabbits compared to the manual method has not been reported previously. We evaluated the performance of the Advia before and after excluding the samples with suboptimal gating in the PEROX cytogram. Abnormal cytograms are considered a trigger for blood smear evaluation because they suggest the presence of underlying leukocyte morphologic abnormalities.^15^ In our study, the vast majority of samples that had suboptimal PEROX gating did not have abnormal leukocyte morphology (only 1 of 12 excluded samples had some pyknotic or karyorrhectic cells). The proportion of abnormal cytograms without morphologic abnormalities (11 of 117 cases; 9.4%) is similar to the proportion of false alerts in dog samples in a previous study.^15^ The exclusion of the samples with suboptimal gating in the PEROX cytogram substantially improved the performance of the Advia for heterophils and eosinophils. This can be primarily attributed to the exclusion of 3 blood samples with unusually high eosinophil percentages in A-Diff (19.9%, 31.4%, and 90.7%; Suppl. Table 1); the M-Diff in all 3 cases revealed that the cells that were classified as eosinophils by the Advia were actually heterophils. A variation in peroxidase staining of eosinophils, as has been documented in humans, dogs, and cats,^6,16,18^ could account for the observed discrepancies between A-Diff and M-Diff in 2 of 3 cases, in which the gating of heterophils and eosinophils appeared suboptimal. In the third sample, the cluster of heterophils was moved toward the right of the PEROX cytogram, possibly indicating increased peroxidase content, which led to the misclassification of heterophils as eosinophils by the Advia. We did not specifically evaluate variation in peroxidase staining of heterophils and eosinophils, but it is an interesting observation that merits further investigation.

The correlation between the 2 methods was very high for heterophils and lymphocytes. The Passing–Bablok regression analyses revealed a small-to-moderate constant error for lymphocytes and a small proportional error for both heterophils and lymphocytes. Additionally, the Bland–Altman analyses revealed a significant negative bias of 9.4% for heterophils and a significant positive bias of 5.5% for lymphocytes between the 2 methods. We repeated analyses after exclusion of samples with marked platelet clumping or toxic changes in heterophils, as these can interfere with leukocyte distinction on Advia PEROX cytograms^15^; however repeated analyses yielded similar results without improving the performance for the 2 leukocyte types, and therefore the source of the observed biases is unclear. The Advia classifies the different leukocyte types, apart from basophils, based on their size and peroxidase content. Given that heterophils and lymphocytes differ in their size and peroxidase content, an inherent inability of the Advia to correctly differentiate the 2 leukocyte populations appears to be unlikely, although it cannot be excluded completely. On the other hand, a possible cause of the observed biases could be the presence of lysed lymphocytes in the evaluated blood smears. Although blood smears of poor quality were excluded from our study, low numbers of lysed leukocytes are inevitably present in every blood smear. Lymphocytes are the most fragile leukocytes^2^; therefore, it is reasonable to assume that some lymphocytes were lysed while preparing the blood smears and were therefore excluded from our manual differential counts. This could have led to an underestimation of lymphocytes (in favor of the predominant population of heterophils) by the 2 observers rather than an overestimation of lymphocytes by the Advia 2120. Nonetheless, the biases between the 2 methods were quite high for several samples, suggesting that, although the previous theory is plausible, it could not account solely for our findings.

A highly important consideration when comparing the results provided by automated hematology analyzers with those obtained manually is that, although the latter is considered the reference method, it is characterized by high variability.^13^ Notably, the value of the quantitative analysis of the manual differential leukocyte counts has been openly questioned as a result of their inherent imprecision, even when > 500 cells are counted.^9^ Therefore, the reported biases for heterophils and lymphocytes could also be associated with the imprecision of the manual differential leukocyte count performed on such a low number of leukocytes compared with the thousands of cells typically evaluated by the Advia. In fact, the CV for manual differential leukocyte counts performed on different days by different observers was 7% for neutrophils and 32% for lymphocytes in one medical study.^4^ The possibility that some heterophils were actually classified as LUCs by the Advia was also considered, but this could not explain the observed magnitude of bias given that LUC percentages were extremely low in our population. A possible effect of sample aging in the performance of the Advia 2120 was also excluded, because blood smears were prepared as soon as the blood samples were received at our laboratory (strictly within 6 h). Finally, uneven leukocyte distribution in the blood smear was an exclusion criterion in our study; however, some blood smears with mildly unevenly distributed leukocytes could have been included in our study, possibly contributing to the observed biases. The reported biases for the 2 leukocyte types should be taken into consideration when evaluating the CBC results obtained with the Advia 2120 given that the inversion of the heterophil-to-lymphocyte ratio is commonly interpreted as an indication of inflammation or corticosteroid-mediated stress.^19^ In particular, it is advisable to use the same method (either manual or automated) when monitoring rabbits with sequential CBCs; the use of method-specific reference intervals in rabbits may also be required based on our results.

The correlation between the 2 methods for basophils was high, and only a minimal constant error and positive bias were observed, which appear unlikely to pose significant clinical implications. Our results indicate that the Advia 2120 can recognize leporine basophils, as suggested previously.^10^ This is in sharp contrast to the well-known inability of the Advia and other automated hematology analyzers to correctly identify canine and feline basophils.^10^

The correlation for monocytes between the 2 methods was moderate. Nonetheless, no significant errors were observed on the Passing–Bablok analysis, and the calculated positive bias was small and likely clinically insignificant. An overestimation of monocyte percentage by automated hematology analyzers, and a moderate or even weak correlation between the automated and manual methods has been reported consistently in the literature, independent of the species and the analyzer used.^11,16,20^ This overestimation can be attributed primarily to the low number of circulating monocytes, which increases the variability of the manual differential leukocyte count. Notably, the CV for manual differential leukocyte counts performed on different days by different observers was as high as 55% for monocytes in one medical study.^4^ Additionally, given that our population also included diseased rabbits with a relatively high occurrence of reactive lymphocytes (noted in almost one-quarter of blood smears), the observed moderate correlation for monocytes could also be related to the potential misclassification of reactive lymphocytes and monocytes by the Advia 2120 or the independent observers. Interestingly, the correlation of monocyte percentages between the 2 observers was not very high (r = 0.800), supporting the inherent imprecision of the manual method and possibly the difficulties in classifying correctly some of the leukocytes when morphologic changes are present.

The correlation between the 2 methods for eosinophils was weak, and a proportional error and a small positive bias were identified. The seemingly poor performance of the Advia 2120 for eosinophils can be attributed to the very low percentages of eosinophils that were detected in our population, similarly to monocytes. It is also noteworthy that the CV for manual differential leukocyte counts performed on different days by different observers was similarly high for eosinophils (69%) as for monocytes in a human medical study.^4^ A variation in peroxidase staining of eosinophils, as has been documented in humans, dogs, and cats,^6,16,18^ was also considered as a possible contributing factor to the observed differences between the 2 methods, but in that case a negative bias would have been expected.

A limitation of our study is that the blood samples were not run through the Advia 2120 in duplicate, as should have been done ideally. However, the limited volume of EDTA-anticoagulated blood precluded such an analysis. A second limitation is that a confident conclusion could not be drawn for eosinophils given the very low percentages observed in our population, but this is an expected finding in rabbits.

## Supplemental Material

sj-pdf-1-vdi-10.1177_10406387211007877 – Supplemental material for Differential white blood cell counts in rabbits: a comparison of the Advia 2120 and a manual method Click here for additional data file. Supplemental material, sj-pdf-1-vdi-10.1177_10406387211007877 for Differential white blood cell counts in rabbits: a comparison of the Advia 2120 and a manual method by Ioannis L. Oikonomidis, Elspeth Milne and Chiara Piccinelli in Journal of Veterinary Diagnostic Investigation
