# Supplementary material for: Differential white blood cell counts in rabbits: a comparison of the Advia 2120 and a manual method
Source: J Vet Diagn Invest. 2021 Apr 9;33(4):670–6. doi: 10.1177/10406387211007877 (PMC8225691; doi:10.1177/10406387211007877)
Supplement: sj-pdf-1-vdi-10.1177_10406387211007877 – Supplemental material for Differential white blood cell counts in rabbits: a comparison of the Advia 2120 and a manual method [file sj-pdf-1-vdi-10.1177_10406387211007877.pdf]

Oikonomidis IL, et al. Differential white blood cell counts in rabbits: a comparison of the Advia 2120 and a manual method

Supplementary Table 1. Raw data of the differential leukocyte counts obtained by the Advia 2120 and the manual method in 104 leporine blood samples; 13 samples were excluded from analysis because of poor blood smear quality. The manual differential leukocyte counts were performed by 2 blinded, independent observers by counting 200 cells in modified Wright-stained blood smears. The mean values obtained from the 2 observers were utilized for the statistical analysis.

Sample	Advia 2120							Mean values of the two observers					Observer 1					Observer 2				
	WBC	NEUT	LYMPH	MONO	EOS	BASO	LUC	NEUT	LYMPH	MONO	EOS	BASO	NEUT	LYMPH	MONO	EOS	BASO	NEUT	LYMPH	MONO	EOS	BASO
1	7.29	71.2	8.5	14.2	2.3	3.4	0.5	78.0	3.5	15.5	0.3	2.8	75.5	5.0	16.5	0.5	2.5	80.5	2.0	14.5	0.0	3.0
2	8.54	39.1	39.6	13.3	2.0	5.2	0.7	49.0	39.5	5.0	0.5	6.0	49.0	37.0	7.0	0.5	6.5	49.0	42.0	3.0	0.5	5.5
3	9.13	48.3	40.0	6.1	0.9	3.8	0.8	67.0	25.5	4.3	0.0	3.3	63.0	28.0	5.5	0.0	3.5	71.0	23.0	3.0	0.0	3.0
4	4.93	48.1	40.4	4.1	2.8	4.5	0.2	59.0	30.8	3.3	1.5	5.5	55.0	34.0	3.5	1.0	6.5	63.0	27.5	3.0	2.0	4.5
5	6.21	60.7	22.0	9.8	3.4	3.5	0.7	65.3	23.5	8.5	1.3	1.5	67.5	22.0	7.5	2.5	0.5	63.0	25.0	9.5	0.0	2.5
6	7.03	31.2	35.2	0.0	31.4	2.2	0.0	56.3	38.0	4.0	1.0	0.8	55.5	36.0	6.5	2.0	0.0	57.0	40.0	1.5	0.0	1.5
7	4.41	51.8	42.7	2.3	1.4	1.6	0.2	68.3	28.3	3.3	0.0	0.3	66.0	31.0	3.0	0.0	0.0	70.5	25.5	3.5	0.0	0.5
8	1.40	41.7	45.4	5.7	6.1	0.5	0.6	56.5	40.0	2.3	0.3	1.0	59.5	36.5	2.5	0.5	1.0	53.5	43.5	2.0	0.0	1.0
9	4.16	39.7	43.7	11.1	2.5	2.5	0.5	50.0	39.3	8.5	0.8	1.5	53.0	35.0	8.5	1.0	2.5	47.0	43.5	8.5	0.5	0.5
10	9.33	31.0	57.1	7.2	1.8	2.2	0.7	36.8	54.0	6.8	0.5	2.0	33.0	58.0	6.5	1.0	1.5	40.5	50.0	7.0	0.0	2.5
11	10.24	41.7	36.6	4.5	0.7	1.7	14.7	56.8	32.8	9.3	0.5	0.8	58.0	34.0	6.5	0.0	1.5	55.5	31.5	12.0	1.0	0.0
12	4.20	50.3	40.9	3.8	1.0	4.0	0.1	62.0	29.3	5.5	0.5	2.8	63.0	29.0	4.5	0.5	3.0	61.0	29.5	6.5	0.5	2.5
13	10.95	22.3	66.7	5.1	2.2	3.4	0.2	29.3	63.8	3.8	0.8	2.5	26.0	68.5	2.0	0.5	3.0	32.5	59.0	5.5	1.0	2.0
14	10.9	31.8	56.2	6.5	1.7	3.3	0.6	39.3	49.8	6.8	1.3	3.0	41.0	48.5	5.5	1.5	3.5	37.5	51.0	8.0	1.0	2.5
15	17.64	73.6	16.5	7.6	0.7	1.3	0.4	75.8	14.8	8.3	0.0	1.3	77.0	15.5	7.0	0.0	0.5	74.5	14.0	9.5	0.0	2.0
16	7.82	20.1	74.4	1.3	1.4	2.6	0.1	24.3	71.5	0.3	0.8	3.3	23.0	72.0	0.5	1.0	3.5	25.5	71.0	0.0	0.5	3.0
17	5.72	38.7	40.4	9.2	6.7	4.0	1.0	47.0	40.5	9.3	0.3	3.0	47.0	43.0	9.0	0.0	1.0	47.0	38.0	9.5	0.5	5.0
18	3.49	56.8	30.9	8.0	3.5	0.6	0.3	76.5	14.0	8.0	0.5	1.0	74.5	16.5	7.5	0.5	1.0	78.5	11.5	8.5	0.5	1.0
19	5.69	24.9	61.9	4.5	3.5	4.0	1.2	46.8	36.3	6.8	4.8	5.5	43.5	36.5	5.5	7.5	7.0	50.0	36.0	8.0	2.0	4.0

20	7.40	35.2	58.6	2.6	1.3	2.1	0.1	37.5	60.5	0.0	1.3	0.8	37.0	62.0	0.0	1.0	0.0	38.0	59.0	0.0	1.5	1.5
21	3.13	69.3	20.6	4.3	2.7	2.8	0.3	73.8	18.5	4.3	0.0	3.5	72.5	19.5	4.0	0.0	4.0	75.0	17.5	4.5	0.0	3.0
22	10.73	1.3	4.6	0.1	90.7	3.3	0.0	83.0	5.8	6.5	0.5	4.3	83.0	6.0	7.0	0.0	4.0	83.0	5.5	6.0	1.0	4.5
23	5.15	39.2	46.8	4.6	1.8	7.2	0.4	57.5	23.3	9.0	1.8	8.5	55.0	24.0	10.5	1.5	9.0	60.0	22.5	7.5	2.0	8.0
24	8.70	34.4	58.1	2.8	0.9	3.2	0.5	46.0	50.0	1.8	0.8	1.5	42.0	54.5	2.0	0.5	1.0	50.0	45.5	1.5	1.0	2.0
25	7.89	34.2	54.1	5.4	0.8	4.7	0.7	49.0	47.0	1.8	0.3	2.0	47.5	50.0	1.0	0.5	1.0	50.5	44.0	2.5	0.0	3.0
26	8.34	38.7	39.0	15.1	0.3	5.8	1.0	44.3	33.3	17.0	0.3	5.3	42.0	34.0	19.5	0.0	4.5	46.5	32.5	14.5	0.5	6.0
27	7.57	50.9	37.2	3.0	2.1	6.6	0.2	62.3	27.0	4.3	2.0	4.5	62.5	26.5	4.0	2.5	4.5	62.0	27.5	4.5	1.5	4.5
28	4.08	64.3	23.4	7.4	2.1	2.3	0.5	74.5	16.5	6.8	0.5	1.8	72.5	15.0	10.0	0.5	2.0	76.5	18.0	3.5	0.5	1.5
29	5.28	62.5	27.3	3.8	2.1	4.0	0.4	70.3	20.8	4.5	1.5	3.0	71.0	19.5	4.0	2.0	3.5	69.5	22.0	5.0	1.0	2.5
30	3.96	42.4	45.3	3.3	2.2	6.6	0.2	54.5	36.8	3.3	0.8	4.8	50.0	40.0	4.5	0.5	5.0	59.0	33.5	2.0	1.0	4.5
31	8.46	58.4	24.7	8.2	2.5	5.8	0.4	63.8	23.5	6.8	0.0	6.0	66.0	22.0	6.5	0.0	5.5	61.5	25.0	7.0	0.0	6.5
32	7.84	29.2	53.0	8.2	1.3	7.8	0.5	37.5	51.0	3.5	1.3	6.8	41.5	46.5	3.5	2.0	6.5	33.5	55.5	3.5	0.5	7.0
33	11.46	50.5	28.0	11.5	3.6	5.6	0.8	51.8	31.3	7.8	3.0	6.3	51.0	30.5	10.0	4.0	4.5	52.5	32.0	5.5	2.0	8.0
34	3.55	44.8	40.6	6.2	2.7	5.1	0.6	63.3	27.3	5.3	0.8	3.5	61.0	31.0	6.0	0.0	2.0	65.5	23.5	4.5	1.5	5.0
35	6.37	48.1	45.2	5.0	0.8	0.5	0.4	54.5	44.0	1.5	0.0	0.0	51.0	47.0	2.0	0.0	0.0	58.0	41.0	1.0	0.0	0.0
36	7.86	59.3	30.0	5.5	1.1	3.4	0.8	64.8	29.3	4.0	0.5	1.5	62.5	31.0	4.5	1.0	1.0	67.0	27.5	3.5	0.0	2.0
37	5.26	60.6	26.9	6.9	1.4	3.9	0.3	67.0	23.3	5.5	0.8	3.5	65.5	23.0	8.0	1.0	2.5	68.5	23.5	3.0	0.5	4.5
38	2.51	47.0	36.5	4.3	4.4	7.3	0.4	54.0	37.3	4.0	0.0	4.8	53.5	38.0	3.5	0.0	5.0	54.5	36.5	4.5	0.0	4.5
39	7.69	42.4	52.0	3.5	0.5	1.0	0.5	58.3	36.0	5.3	0.3	0.3	56.5	39.0	3.5	0.5	0.5	60.0	33.0	7.0	0.0	0.0
40	8.08	63.3	27.8	6.0	0.8	1.3	0.8	76.5	16.0	6.5	0.3	0.8	76.0	18.5	5.0	0.0	0.5	77.0	13.5	8.0	0.5	1.0
41	6.16	83.1	6.8	5.6	2.2	2.1	0.3	89.0	6.5	2.5	0.5	1.5	88.5	6.0	2.5	0.5	2.5	89.5	7.0	2.5	0.5	0.5
42	5.60	40.9	45.9	8.3	1.3	3.2	0.4	53.8	35.8	6.3	1.8	2.5	52.5	37.5	5.0	1.5	3.5	55.0	34.0	7.5	2.0	1.5
43	5.38	43.5	42.3	6.0	1.3	5.2	1.5	59.8	31.8	2.0	0.3	6.3	59.5	31.0	3.0	0.0	6.5	60.0	32.5	1.0	0.5	6.0
44	3.61	51.1	33.4	8.8	1.2	5.2	0.3	54.3	34.5	6.8	0.0	4.5	51.0	37.5	6.5	0.0	5.0	57.5	31.5	7.0	0.0	4.0
45	5.53	36.3	58.9	1.5	0.3	2.7	0.1	44.0	52.3	2.0	0.3	1.5	44.5	52.0	2.0	0.5	1.0	43.5	52.5	2.0	0.0	2.0
46	10.38	23.3	69.7	4.9	0.4	1.4	0.4	30.8	67.0	1.8	0.0	0.5	29.0	68.5	1.5	0.0	1.0	32.5	65.5	2.0	0.0	0.0
47	8.51	69.1	16.3	5.9	2.4	5.3	1.0	81.5	9.5	4.5	0.3	4.3	80.5	9.5	4.5	0.5	5.0	82.5	9.5	4.5	0.0	3.5
48	6.77	58.8	34.3	4.2	0.9	1.2	0.6	76.3	17.3	4.8	1.0	0.8	76.5	17.0	5.5	1.0	0.0	76.0	17.5	4.0	1.0	1.5
49	6.96	37.0	57.2	2.9	0.3	2.0	0.6	42.8	52.3	4.5	0.0	0.5	41.5	53.0	5.5	0.0	0.0	44.0	51.5	3.5	0.0	1.0
50	2.74	49.3	43.1	2.2	1.4	4.0	0.1	60.5	30.5	5.5	0.0	3.5	62.5	29.5	5.5	0.0	2.5	58.5	31.5	5.5	0.0	4.5
51	7.15	32.2	49.1	10.0	1.5	6.8	0.4	35.8	45.3	10.8	0.0	8.3	35.5	45.5	10.0	0.0	9.0	36.0	45.0	11.5	0.0	7.5
52	3.70	65.9	21.5	7.5	1.7	2.9	0.5	70.5	20.8	4.8	0.5	3.5	68.5	23.0	5.0	0.5	3.0	72.5	18.5	4.5	0.5	4.0

53	6.27	38.3	44.1	1.0	10.5	6.0	0.1	44.5	42.0	1.0	9.0	3.5	46.0	42.5	1.0	8.5	2.0	43.0	41.5	1.0	9.5	5.0
54	5.61	9.9	86.0	1.0	0.9	2.0	0.3	40.0	56.8	1.5	1.5	0.3	40.0	57.0	2.0	1.0	0.0	40.0	56.5	1.0	2.0	0.5
55	4.20	44.7	45.1	5.1	1.2	2.0	2.0	53.3	38.5	6.3	0.5	1.5	54.0	37.5	8.0	0.5	0.0	52.5	39.5	4.5	0.5	3.0
56	7.15	77.6	8.5	9.9	1.5	1.2	1.4	85.0	5.5	8.3	0.8	0.5	86.0	5.0	8.0	1.0	0.0	84.0	6.0	8.5	0.5	1.0
57	3.64	22.7	73.1	1.8	0.5	1.1	0.9	30.8	64.3	3.8	0.5	0.8	29.5	65.0	4.5	0.0	1.0	32.0	63.5	3.0	1.0	0.5
58	8.05	59.8	28.2	5.5	2.0	4.1	0.4	59.0	33.0	2.5	0.0	5.5	57.0	36.0	3.0	0.0	4.0	61.0	30.0	2.0	0.0	7.0
59	6.26	26.0	48.7	11.6	3.7	9.2	0.7	26.5	53.5	11.0	0.0	9.0	28.0	54.0	9.0	0.0	9.0	25.0	53.0	13.0	0.0	9.0
60	3.99	38.7	43.2	12.3	2.0	3.7	0.1	44.8	46.0	5.5	0.5	3.3	43.5	47.0	5.5	0.5	3.5	46.0	45.0	5.5	0.5	3.0
61	5.75	34.1	53.3	7.2	2.1	3.1	0.2	37.3	53.8	5.0	2.3	1.8	40.5	50.5	6.0	2.5	0.5	34.0	57.0	4.0	2.0	3.0
62	3.97	49.5	38.7	3.2	2.3	6.0	0.3	65.0	27.5	2.8	1.3	3.5	62.5	30.5	3.0	0.5	3.5	67.5	24.5	2.5	2.0	3.5
63	6.12	55.2	31.9	5.3	1.4	6.1	0.1	65.8	21.3	5.3	1.0	6.8	67.0	20.5	6.0	0.5	6.0	64.5	22.0	4.5	1.5	7.5
64	8.81	45.3	31.5	14.0	1.8	6.5	1.0	48.0	30.8	13.5	0.5	7.3	49.0	28.5	13.0	0.5	9.0	47.0	33.0	14.0	0.5	5.5
65	8.50	27.6	61.9	4.1	1.9	4.5	0.0	28.3	64.0	3.3	1.3	3.3	25.0	66.0	4.0	1.5	3.5	31.5	62.0	2.5	1.0	3.0
66	10.54	65.4	23.8	6.1	0.9	2.3	1.3	65.3	28.0	5.5	0.0	1.3	65.5	29.0	5.0	0.0	0.5	65.0	27.0	6.0	0.0	2.0
67	3.95	34.4	54.8	6.8	1.2	2.4	0.4	39.0	56.0	2.3	0.8	2.0	37.0	58.0	2.5	1.0	1.5	41.0	54.0	2.0	0.5	2.5
68	6.50	84.1	9.0	5.3	0.9	0.5	0.1	91.0	5.8	3.0	0.0	0.3	91.0	6.0	3.0	0.0	0.0	91.0	5.5	3.0	0.0	0.5
69	2.82	38.9	52.2	5.0	2.7	0.9	0.2	45.0	51.5	2.5	0.0	1.0	42.5	55.0	1.5	0.0	1.0	47.5	48.0	3.5	0.0	1.0
70	4.33	50.7	33.4	10.9	1.3	3.2	0.5	57.3	32.3	9.0	0.5	1.0	57.0	32.5	7.5	1.0	2.0	57.5	32.0	10.5	0.0	0.0
71	3.88	64.3	23.6	2.1	2.2	7.8	0.1	74.5	12.8	2.5	1.8	8.5	78.5	12.0	3.0	0.5	6.0	70.5	13.5	2.0	3.0	11.0
72	4.02	60.0	19.4	6.9	9.9	3.5	0.3	73.3	20.3	6.0	0.0	0.5	73.0	23.5	3.0	0.0	0.5	73.5	17.0	9.0	0.0	0.5
73	9.15	57.9	25.6	9.8	0.5	5.1	1.2	55.5	33.8	7.3	0.0	3.5	54.0	35.0	7.0	0.0	4.0	57.0	32.5	7.5	0.0	3.0
74	2.92	62.7	21.8	10.8	1.1	3.0	0.6	74.3	17.5	5.0	0.0	3.3	75.5	19.0	3.0	0.0	2.5	73.0	16.0	7.0	0.0	4.0
75	4.39	46.7	45.7	2.0	0.8	4.5	0.3	71.3	22.8	1.8	0.3	4.0	77.5	18.5	1.0	0.0	3.0	65.0	27.0	2.5	0.5	5.0
76	4.11	30.6	61.5	1.8	2.2	3.7	0.2	56.0	39.3	0.8	0.3	3.8	60.0	36.0	0.5	0.0	3.5	52.0	42.5	1.0	0.5	4.0
77	3.65	49.5	40.4	1.2	1.4	7.3	0.2	79.0	16.8	1.0	0.5	2.8	77.5	18.5	1.0	0.5	2.5	80.5	15.0	1.0	0.5	3.0
78	2.63	39.3	48.6	4.8	2.1	4.8	0.4	48.8	43.8	1.3	1.0	5.3	50.0	44.0	0.5	0.5	5.0	47.5	43.5	2.0	1.5	5.5
79	6.54	32.8	50.2	4.8	1.9	10.0	0.3	41.3	45.8	2.5	1.8	8.8	36.0	52.5	2.5	1.5	7.5	46.5	39.0	2.5	2.0	10.0
80	6.81	85.4	9.5	2.2	0.8	1.6	0.5	92.3	5.8	1.5	0.0	0.5	90.5	6.5	2.0	0.0	1.0	94.0	5.0	1.0	0.0	0.0
81	5.57	46.9	44.3	2.2	1.4	5.2	0.1	61.8	26.8	3.8	1.0	6.8	60.5	27.0	4.5	1.0	7.0	63.0	26.5	3.0	1.0	6.5
82	4.75	71.7	21.0	2.1	1.8	2.7	0.7	91.3	6.5	0.0	0.5	1.8	88.5	9.5	0.0	0.5	1.5	94.0	3.5	0.0	0.5	2.0
83	3.92	35.0	50.7	6.9	3.3	4.0	0.1	49.0	47.8	1.0	0.5	1.8	46.0	51.5	1.5	0.0	1.0	52.0	44.0	0.5	1.0	2.5
84	2.52	35.2	50.2	3.2	2.2	8.7	0.5	51.8	47.3	0.5	0.0	0.5	51.0	48.0	0.5	0.0	0.5	52.5	46.5	0.5	0.0	0.5
85	6.10	70.2	17.6	8.3	0.8	2.6	0.5	74.8	18.3	4.0	0.5	2.5	73.0	21.0	4.0	0.5	1.5	76.5	15.5	4.0	0.5	3.5

86	4.36	42.4	48.0	4.5	0.9	4.0	0.1	62.8	29.5	3.5	0.3	4.0	60.0	32.5	4.0	0.0	3.5	65.5	26.5	3.0	0.5	4.5
87	6.63	54.3	24.7	10.4	1.6	8.3	0.6	59.8	26.0	2.5	0.8	11.0	58.5	28.0	2.5	1.5	9.5	61.0	24.0	2.5	0.0	12.5
88	9.43	40.1	39.8	15.1	1.2	3.2	0.6	48.5	40.0	7.5	0.8	3.3	56.5	32.5	6.5	0.5	4.0	40.5	47.5	8.5	1.0	2.5
89	1.85	57.2	32.9	4.1	1.6	4.2	0.1	69.8	20.8	4.3	0.5	4.8	67.5	23.5	5.5	0.5	3.0	72.0	18.0	3.0	0.5	6.5
90	17.47	79.8	9.7	4.0	2.6	3.7	0.3	88.5	6.8	3.5	0.3	1.0	86.5	8.5	3.0	0.0	2.0	90.5	5.0	4.0	0.5	0.0
91	3.23	46.8	34.4	8.8	2.9	7.0	0.1	67.5	22.5	2.8	2.0	5.3	69.5	21.0	3.0	1.5	5.0	65.5	24.0	2.5	2.5	5.5
92	10.29	81.1	11.0	4.9	1.3	1.4	0.3	92.3	3.5	3.8	0.0	0.5	90.0	4.0	6.0	0.0	0.0	94.5	3.0	1.5	0.0	1.0
93	7.82	49.9	36.7	7.1	1.2	4.9	0.3	61.3	29.8	1.5	0.0	7.5	60.5	31.5	1.0	0.0	7.0	62.0	28.0	2.0	0.0	8.0
94	3.65	45.2	45.9	0.9	3.2	4.7	0.1	56.8	36.5	2.3	0.3	4.3	54.5	41.5	1.0	0.0	3.0	59.0	31.5	3.5	0.5	5.5
95	7.01	47.5	32.0	6.7	6.9	6.6	0.2	63.3	27.3	1.0	3.3	5.3	63.5	28.0	0.5	3.5	4.5	63.0	26.5	1.5	3.0	6.0
96	5.43	48.1	45.6	1.3	1.2	3.7	0.1	70.5	23.5	1.8	0.0	4.3	70.0	23.5	1.5	0.0	5.0	71.0	23.5	2.0	0.0	3.5
97	5.12	23.1	52.4	0.0	19.9	4.5	0.0	46.0	48.5	1.5	1.0	3.0	42.5	53.5	0.0	1.0	3.0	49.5	43.5	3.0	1.0	3.0
98	5.45	70.8	7.9	11.4	3.7	5.8	0.5	79.5	2.5	12.3	0.3	5.5	80.5	2.0	12.0	0.0	5.5	78.5	3.0	12.5	0.5	5.5
99	8.53	60.8	26.5	5.0	1.8	5.8	0.1	79.8	16.5	1.5	0.5	1.8	81.0	15.0	1.0	0.5	2.5	78.5	18.0	2.0	0.5	1.0
100	6.80	33.7	52.5	7.8	2.0	3.4	0.7	54.8	34.0	8.3	0.5	2.5	53.0	36.5	7.5	0.0	3.0	56.5	31.5	9.0	1.0	2.0
101	4.91	39.7	42.4	4.5	5.2	8.0	0.3	47.5	42.0	2.0	0.5	8.0	46.5	42.0	2.0	0.5	9.0	48.5	42.0	2.0	0.5	7.0
102	3.82	30.3	59.8	4.7	3.9	1.2	0.1	34.3	61.5	2.5	0.0	1.8	33.0	62.5	2.5	0.0	2.0	35.5	60.5	2.5	0.0	1.5
103	6.65	47.3	40.8	10.0	0.9	0.8	0.4	55.0	36.3	8.0	0.0	0.8	55.0	39.0	5.0	0.0	1.0	55.0	33.5	11.0	0.0	0.5
104	2.06	26.8	57.7	3.0	4.5	7.4	0.6	20.3	73.0	2.3	1.3	3.3	17.0	76.0	2.0	1.5	3.5	23.5	70.0	2.5	1.0	3.0

Red indicates suboptimal gating of the leukocytes in the Advia peroxidase cytogram. BASO = basophil percentage; EOS = eosinophil percentage; LUC = large unstained cell percentage; LYMPH = lymphocyte percentage; MONO = monocyte percentage; NEUT = neutrophil percentage; WBC = white blood cells (× 10^9/L).

Supplementary Table 2. Descriptive statistics and results of the correlation study, Passing–Bablok, and Bland–Altman analyses comparing the differential leukocyte counts obtained by the Advia 2120 and the manual method in 104 leporine blood samples. Thirteen samples were excluded previously from analysis because of poor blood smear quality. The manual differential leukocyte counts were performed by 2 blinded, independent observers by counting 200 cells in modified Wright-stained blood smears. The mean values obtained from the 2 observers were utilized for the statistical analysis.

Leukocyte	Advia mean/median*	Manual mean/median*	Correlation coefficient (*p*)	Bias†	Lower limit of bias†	Upper limit of bias†	Estimated intercept†	Estimated slope†
Heterophils	47.2% (16.4%)	58.3% (16.5%)	0.822 (<0.001)	–11.2 (–13.0, –9.2)	–30.4 (–33.7, –27.1)	8.1 (4.8, 11.4)	–6.3 (–12.1, –2.32)	0.95 (0.86, 1.03)
Lymphocytes	39.0% (16.5%)	33.0% (16.6%)	0.891 (<0.001)	6.0 (4.5, 7.5)	–9.2 (–11.8, –6.6)	21.1 (18.6, 23.8)	5.2 (2.6, 8.4)	0.98 (0.91, 1.05)
Monocytes	5.2% (0.0–15.1%)	4.0% (0.0–17.0%)	0.624 (<0.001)	1.2 (0.7, 1.7)	–4.0 (–4.8, –3.1)	6.3 (5.4, 7.2)	0.52 (–0.50, 1.33)	1.10 (0.95, 1.34)
Eosinophils	1.8% (0.3–90.7%)	0.5% (0.0–9.0%)	0.289 (0.003)	2.7 (0.8, 4.5)	–15.7 (–18.9, –12.6)	21.1 (17.9, 24.3)	0.20 (–2.31, 0.76)	3.20 (1.75, 10.44)
Basophils	3.7% (0.5–10.0%)	3.0% (0.0–11.0%)	0.814 (<0.001)	0.7 (0.4, 1.0)	–2.2 (–2.7, –1.7)	3.6 (3.1, 4.1)	0.89 (0.57, 1.23)	0.90 (0.80, 1.02)

* Mean (SD) is reported for neutrophils and lymphocytes (Gaussian data distribution according to Shapiro–Wilk test), and median (range) is reported for monocytes, eosinophils, and basophils (non-Gaussian data distribution according to Shapiro–Wilk test).

† Numbers in parentheses are 95% confidence intervals.
